# The Development, Differentiation, and Toxicity in Reproduction

**DOI:** 10.3390/ijms23137183

**Published:** 2022-06-28

**Authors:** Byeongseok Kim, Youngsok Choi

**Affiliations:** 1 Department of Stem Cell and Regenerative Biotechnology, Konkuk University, Seoul 05029, Korea; qufsksrlaqud@naver.com; 2 Humanized Pig Center, Konkuk University, Seoul 05029, Korea

This Special Issue is intended to provide up-to-date information on reproduction, including the reproduction of germ cells and reproductive organs (ovary, testis, and uterus). Reproduction is an important process, entailing passing on one’s genetic information to the next generation. Recently, the infertility rate has been increasing worldwide. A number of factors are involved in this, such as genetic disorders and the environment. For this reason, understanding reproductive physiology is important. We applaud the scientists who continue to study this topic.

We looked at how many recent studies were being conducted in the field of reproduction through Pubmed (http://pubmed.ncbi.nlm.nih.gov). As of 2021, 4035 papers were published on testes, 5496 on sperm, 4512 on ovaries, 3690 on oocytes, and 4512 papers on the uterus (Table 1). 

Of course, there will be some duplicates, and there will be articles missing from the search. However, this area is relatively lacking in number compared to other hot research fields, such as the gut microbiome (10,105 articles). Compared to 10 years ago, these figures on the literature on reproduction have only shown an increase of 33% on average. On the other hand, the gut microbiome showed a phenomenal increase of 692% (Table 1).

This relatively small number does not mean that the field is unimportant. As many studies are examined, there is the possibility of a breakthrough that can solve the difficult problems in this area.

When we were first proposed to create a Special Issue on this topic with the MDPI group, we were a little hesitant because we were not sure whether we would be able to obtain related papers. However, contrary to what we were worried about, the response was great. Therefore, we selected the 25 most valuable studies in this Special Issue.

First of all, we appreciate all the researchers who passionately carried out their studies and the referees who reviewed the manuscripts, even during difficult times due to the COVID-19 pandemic. By looking at the content of reproductive research, it was possible to determine its trends. Among the selected papers, about half were related to male reproduction, e.g., testis and sperm, [1,2,3,4,5,6,7,8,9,10,11,12], and the other half were associated with female reproduction, e.g., oocytes, as well as embryos and their implantation, including three reviews [13,14,15,16,17,18,19,20,21,22,23,24,25]. 

Eight papers were reported on oocytes and embryos; the studies of Park et al. (2020), Jeong et al. (2020), and Kim et al. (2020) were designed to investigate the effects of antimicrobial agents, triclosan and butyl parabens on embryonic and embryonic cells [15,16,23]. In particular, Park and colleagues suggested that pig eggs damaged by triclosan exposure can be rescued using specific scavengers [23]. Kim et al. reported that this induces DNA damage and mitochondrial dysfunction and interferes with the implantation of porcine embryos exposed to it [16]. Another antimicrobial agent, butylparaben, can also cause mitochondrial abnormalities through ROS production, which is also believed to affect the maturation of eggs and embryo development after in vitro fertilization [15]. Additionally, Mesalam et al. reported on the effect of juglone exposure on oocyte development through the induction of oxidative stress and mitochondrial dysfunction [20]. The use of these antimicrobial agents means that exposure to these substances can cause infertility, so related results can be used as an important data to identify the cause of infertility as well as understand the early development of embryos in the future. 

Interestingly, in this Special Issue, four articles reported on the effects of environmental hormones, bisphenol-A (BPA) and nonylphenol, on sperm development. Lombo et al. reported the effect of BPA on sperm in zebrafish males and observed abnormal spermatogenesis and epigenetics in the progenies of parents exposed to BPA [7]. Dr. Song’s group examined the effect of nonylphenol on mouse testes and reported that NP-exposed testis had problems with spermatogenesis due to an abnormal hormonal balance and the induction of apoptosis [10]. This means that the use of environmental hormones is increasing due to recent industrial developments, and the fact that they affect not only our health but also the fertility of our offspring in the future should not be overlooked. 

In addition to germ cells, three papers on the uterus were also published in this Special Issue. The uterus tissue periodically undergoes dynamic changes as well as the decidualization of uterine endometrium to initiate a successful pregnancy. This involves a morphological and functional differentiation of endometrial stromal cells. Yang et al. reported that laminin A5 is involved in decidualization through the PKA-CCAAT enhancer-binding protein β (C/EBPβ) signaling pathway under progesterone [24]. In fact, the dynamic regulation(s) of uterine endometrium are precisely regulated by two main hormones: estrogen and progesterone. Additionally, their regulation involves several cellular signaling pathway along with these hormonal signaling pathways. Recently, several researchers, including us, have been studying the role of hippo signaling in the uterus [21]. In this review, we describe the relationship between hormonal regulation and the hippo signaling pathway as well as a novel regulatory mechanism in the expression of target genes in the uterine endometrium.

There are additional areas mentioned in this Special Issue, such as results characterizing factors important to the development and function of germ cells and embryos. Various factors such as pigment epithelium-derived factor (PEDF) [1], Brahma-related gene 1 (BRG1) [12], Zinc finger Asp-His-His-Cys palmitoyltranferase 19 (ZDHHC 19) [11], BAF-L (Barrier-like protein) [4], Mitofusin 1 (MFN1), and MFN2 [8] are reported on, with their roles in spermatogenesis and spermiogenesis being highlighted. Yoon et al. and Oh et al. reported on the roles and molecular mechanisms of Peroxisome proliferator-activated receptor gamma (PPARγ) [25] and Disabled homolog 2 (DAB2) [22] in the development of primordial follicles and porcine embryos, respectively. Bogolyubova and Bogolyubov reviewed the role of Death-domain associated protein 6 (DAXX) in the development of oogenesis and early embryogenesis [13].

La et al. applied a single-cell transcriptomic analysis, a cutting-edge technique for understanding germ cell formation [18]. To analyze a cell’s genetic profile, a total RNA extract of the tissue is usually used. However, since the cells present in these tissues are in a heterogeneous state, in which several types are mixed, it is necessary to purely isolate the target cells from the tissues. Additionally, even if only the desired cells are isolated from tissues, a certain number of cells must be secured for analysis. In this respect, a single-cell transcriptomic analysis is suitable for germ cells, including oocytes and sperms. 

Finally, we would like to thank all the reproductive biologists who are contributing research from various perspectives in the reproductive field in this difficult time due to COVID-19. We have opened another Special Issue following this Special Issue entitled “Biology and Toxicology of Gametes, Embryos, and Cancer Cells in Reproductive System”. We encourage you to share your valuable results with continued interest and contribution in the future.

## Figures and Tables

**Table 1:** Number of articles searched based on keywords in Pubmed.

Keyword	Year		Increase Rate
	2011	2021	
Testis	3383	4035	19%
Sperm	3950	5496	39%
Ovary	4872	6226	28%
Oocyte	3031	4512	49%
Uterus	3451	4512	30%
Gut Microbiome	144	10,105	692%

## References

[1] Bagdadi N., Sawaied A., AbuMadighem A., Lunenfeld E., Huleihel M. (2021). The Expression Levels and Cellular Localization of Pigment Epithelium Derived Factor (PEDF) in Mouse Testis: Its Possible Involvement in the Differentiation of Spermatogonial Cells. Int. J. Mol. Sci..

[2] Cazin C., Boumerdassi Y., Martinez G., Fourati Ben Mustapha S., Whitfield M., Coutton C., Thierry-Mieg N., Di Pizio P., Rives N., Arnoult C. (2021). Identification and Characterization of the Most Common Genetic Variant Responsible for Acephalic Spermatozoa Syndrome in Men Originating from North Africa. Int. J. Mol. Sci..

[3] Chioccarelli T., Migliaccio M., Suglia A., Manfrevola F., Porreca V., Diano N., Errico S., Fasano S., Cobellis G. (2021). Characterization of Estrogenic Activity and Site-Specific Accumulation of Bisphenol-A in Epididymal Fat Pad: Interfering Effects on the Endocannabinoid System and Temporal Progression of Germ Cells. Int. J. Mol. Sci..

[4] Huang C., Gong H., Mu B., Lan X., Yang C., Tan J., Liu W., Zou Y., Li L., Feng B. (2022). BAF-L Modulates Histone-to-Protamine Transition during Spermiogenesis. Int. J. Mol. Sci..

[5] Karmakar P.C., Ahn J.S., Kim Y.H., Jung S.E., Kim B.J., Lee H.S., Kim S.U., Rahman M.S., Pang M.G., Ryu B.Y. (2020). Paternal Exposure to Bisphenol-A Transgenerationally Impairs Testis Morphology, Germ Cell Associations, and Stemness Properties of Mouse Spermatogonial Stem Cells. Int. J. Mol. Sci..

[6] Karmakar P.C., Ahn J.S., Kim Y.H., Jung S.E., Kim B.J., Lee H.S., Ryu B.Y. (2020). Gestational Exposure to Bisphenol A Affects Testicular Morphology, Germ Cell Associations, and Functions of Spermatogonial Stem Cells in Male Offspring. Int. J. Mol. Sci..

[7] Lombo M., Herraez M.P. (2021). Paternal Inheritance of Bisphenol A Cardiotoxic Effects: The Implications of Sperm Epigenome. Int. J. Mol. Sci..

[8] Miao J., Chen W., Wang P., Zhang X., Wang L., Wang S., Wang Y. (2021). MFN1 and MFN2 Are Dispensable for Sperm Development and Functions in Mice. Int. J. Mol. Sci..

[9] Park H.J., Lee R., Yoo H., Hong K., Song H. (2020). Nonylphenol Induces Apoptosis through ROS/JNK Signaling in a Spermatogonia Cell Line. Int. J. Mol. Sci..

[10] Park H.J., Zhang M., Lee W.Y., Hong K.H., Do J.T., Park C., Song H. (2020). Toxic Effects of Nonylphenol on Neonatal Testicular Development in Mouse Organ Culture. Int. J. Mol. Sci..

[11] Wang S., Qiao H., Wang P., Wang Y., Qin D. (2021). ZDHHC19 Is Dispensable for Spermatogenesis, but Is Essential for Sperm Functions in Mice. Int. J. Mol. Sci..

[12] Wang S., Wang P., Liang D., Wang Y. (2020). BRG1 Is Dispensable for Sertoli Cell Development and Functions in Mice. Int. J. Mol. Sci..

[13] Bogolyubova I., Bogolyubov D. (2021). DAXX Is a Crucial Factor for Proper Development of Mammalian Oocytes and Early Embryos. Int. J. Mol. Sci..

[14] Drzewiecka E.M., Kozlowska W., Paukszto L., Zmijewska A., Wydorski P.J., Jastrzebski J.P., Franczak A. (2021). Effect of the Electromagnetic Field (EMF) Radiation on Transcriptomic Profile of Pig Myometrium during the Peri-Implantation Period-An In Vitro Study. Int. J. Mol. Sci..

[15] Jeong P.S., Lee S., Park S.H., Kim M.J., Kang H.G., Nanjidsuren T., Son H.C., Song B.S., Koo D.B., Sim B.W. (2020). Butylparaben Is Toxic to Porcine Oocyte Maturation and Subsequent Embryonic Development Following In Vitro Fertilization. Int. J. Mol. Sci..

[16] Kim M.J., Park H.J., Lee S., Kang H.G., Jeong P.S., Park S.H., Park Y.H., Lee J.H., Lim K.S., Lee S.H. (2020). Effect of Triclosan Exposure on Developmental Competence in Parthenogenetic Porcine Embryo during Preimplantation. Int. J. Mol. Sci..

[17] Kulus M., Kranc W., Wojtanowicz-Markiewicz K., Celichowski P., Swiatly-Blaszkiewicz A., Matuszewska E., Sujka-Kordowska P., Konwerska A., Zdun M., Bryl R. (2021). New Gene Markers Expressed in Porcine Oviductal Epithelial Cells Cultured Primary In Vitro Are Involved in Ontological Groups Representing Physiological Processes of Porcine Oocytes. Int. J. Mol. Sci..

[18] La H., Yoo H., Lee E.J., Thang N.X., Choi H.J., Oh J., Park J.H., Hong K. (2021). Insights from the Applications of Single-Cell Transcriptomic Analysis in Germ Cell Development and Reproductive Medicine. Int. J. Mol. Sci..

[19] Lee J., Park H., Moon S., Do J.T., Hong K., Choi Y. (2021). Expression and Regulation of CD73 during the Estrous Cycle in Mouse Uterus. Int. J. Mol. Sci..

[20] Mesalam A.A., El-Sheikh M., Joo M.D., Khalil A.A.K., Mesalam A., Ahn M.J., Kong I.K. (2020). Induction of Oxidative Stress and Mitochondrial Dysfunction by Juglone Affects the Development of Bovine Oocytes. Int. J. Mol. Sci..

[21] Moon S., Hwang S., Kim B., Lee S., Kim H., Lee G., Hong K., Song H., Choi Y. (2022). Hippo Signaling in the Endometrium. Int. J. Mol. Sci..

[22] Oh J.N., Lee M., Choe G.C., Lee D.K., Choi K.H., Kim S.H., Jeong J., Lee C.K. (2020). Identification of the Lineage Markers and Inhibition of DAB2 in In Vitro Fertilized Porcine Embryos. Int. J. Mol. Sci..

[23] Park H.J., Song B.S., Kim J.W., Yang S.G., Kim S.U., Koo D.B. (2020). Exposure of Triclosan in Porcine Oocyte Leads to Superoxide Production and Mitochondrial-Mediated Apoptosis During In Vitro Maturation. Int. J. Mol. Sci..

[24] Yang Z.S., Pan H.Y., Shi W.W., Chen S.T., Wang Y., Li M.Y., Zhang H.Y., Yang C., Liu A.X., Yang Z.M. (2021). Regulation and Function of Laminin A5 during Mouse and Human Decidualization. Int. J. Mol. Sci..

[25] Yoon S.Y., Kim R., Jang H., Shin D.H., Lee J.I., Seol D., Lee D.R., Chang E.M., Lee W.S. (2020). Peroxisome Proliferator-Activated Receptor Gamma Modulator Promotes Neonatal Mouse Primordial Follicle Activation In Vitro. Int. J. Mol. Sci..

